# NIK Stabilization in Osteoclasts Results in Osteoporosis and Enhanced Inflammatory Osteolysis

**DOI:** 10.1371/journal.pone.0015383

**Published:** 2010-11-08

**Authors:** Chang Yang, Kathleen McCoy, Jennifer L. Davis, Marc Schmidt-Supprian, Yoshiteru Sasaki, Roberta Faccio, Deborah Veis Novack

**Affiliations:** 1 Division of Bone and Mineral Diseases, Department of Medicine, Washington University School of Medicine, St. Louis, Missouri, United States of America; 2 Department of Pathology, Washington University School of Medicine, St. Louis, Missouri, United States of America; 3 Department of Orthopedic Surgery, Washington University School of Medicine, St. Louis, Missouri, United States of America; 4 Max Planck Institute of Biochemistry, Martinsried, Germany; 5 RIKEN Center for Developmental Biology, Kobe, Japan; University of California Los Angeles and Cedars-Sinai Medical Center, United States of America

## Abstract

**Background:**

Maintenance of healthy bone requires the balanced activities of osteoclasts (OCs), which resorb bone, and osteoblasts, which build bone. Disproportionate action of OCs is responsible for the bone loss associated with postmenopausal osteoporosis and rheumatoid arthritis. NF-κB inducing kinase (NIK) controls activation of the alternative NF-κB pathway, a critical pathway for OC differentiation. Under basal conditions, TRAF3-mediated NIK degradation prevents downstream signaling, and disruption of the NIK:TRAF3 interaction stabilizes NIK leading to constitutive activation of the alternative NF-κB pathway.

**Methodology/Principal Findings:**

Using transgenic mice with OC-lineage expression of NIK lacking its TRAF3 binding domain (NT3), we now find that alternative NF-κB activation enhances not only OC differentiation but also OC function. Activating NT3 with either lysozyme M Cre or cathepsinK Cre causes high turnover osteoporosis with increased activity of OCs and osteoblasts. In vitro, NT3-expressing precursors form OCs more quickly and at lower doses of RANKL. When cultured on bone, they exhibit larger actin rings and increased resorptive activity. OC-specific NT3 transgenic mice also have an exaggerated osteolytic response to the serum transfer model of arthritis.

**Conclusions:**

Constitutive activation of NIK drives enhanced osteoclastogenesis and bone resorption, both in basal conditions and in response to inflammatory stimuli.

## Introduction

Osteoclasts (OCs) are the only cells capable of bone resorption, a process required for both normal bone homeostasis and pathological bone loss [1]. These terminally differentiated, multinucleated cells are derived from precursors in the monocyte/macrophage lineage. The primary cytokine mediating OC differentiation is receptor activator of NF-κB ligand (RANKL), a member of the TNF superfamily. RANKL, working via its receptor RANK, commits early precursors to the OC fate, and causes fusion of these preosteoclasts to generate mature multinucleated cells. OCs attach to the bone surface, via αvβ3 integrins, forming a tight sealing zone that delineates a resorptive lacuna into which acid and matrix-degrading enzymes are secreted [2]. The actin ring is a distinctive cytoskeletal structure that OCs must form in order to generate a sealing zone. Many signaling pathways, including those downstream of RANKL, appear to contribute to actin ring formation, but specific transcriptional programs have not been defined.

Even before the identification of RANKL, NF-κB was identified as an important pathway in the context of bone when it was found that mice lacking both the p50 and p52 subunits were osteopetrotic, with a complete absence of OCs [3], [4]. More recent studies have defined two distinct NF-κB pathways, both of which are activated by RANKL in osteoclast lineage cells [5]. The primary role of the classical pathway is to allow survival of OC precursors [6], [7]. In contrast, the alternative or non-canonical NF-κB pathway controls OC differentiation, but not survival [8], [9]. It is initiated by the upstream kinase NIK, and culminates in transcription of target genes by RelB/p52 NF-κB dimers. This pathway is negatively regulated at 2 levels, by the instability of NIK protein and the retention of RelB in the cytoplasm by p100. In unstimulated cells, NIK interacts with TRAF3, leading to ubiquitination by cIAPs and degradation by the proteosome, keeping total cellular NIK levels very low [10], [11]. Upon RANKL stimulation, TRAF3 is degraded and NIK is stabilized in the cell. NIK then promotes processing of p100 to p52 by the proteosome, leading to accumulation of active RelB/p52 dimers in the nucleus.

We have previously shown that absence of NIK or RelB in OCs blocks osteoclastogenesis, in vitro, and pathological osteolysis in the context of inflammation and bone metastasis, but has little effect on basal bone homeostasis [8], [9], [12]. However, in these studies utilizing the globally NIK-deficient mouse, the complete lack of OC differentiation, in vitro, and the effect of NIK deletion in other cell types, in vivo, limited our ability to fully delineate the role of NIK and the alternative NF-κB pathway in the OC lineage.

Recently, constitutive activation of NIK – by direct mutation or mutation of its negative regulators cIAP1/2 and TRAF3 – has been identified in multiple myeloma [13], [14]. This aberrant NIK activation leads to increased cell survival and proliferation of malignant plasma cells. Although it did not cause myeloma in mice, transgenic expression of a constitutively active NIK in B cells caused growth factor independent B cell hyperplasia [15]. This constitutively active NIK allele – NIKΔT3– lacks the TRAF3 binding domain, preventing the degradation that normally keeps NIK levels low in resting cells. Using mice expressing this mutant NIK allele in OC lineage cells, we describe the effects of constitutive NIK activation in OCs both in vivo and in vitro. We find that NIKΔT3 transgenic mice are osteoporotic at baseline, and are much more sensitive to inflammatory osteolysis than nontransgenic littermates using the serum transfer model of arthritis. In vitro, NIKΔT3 drives more robust OC differentiation and generates more active OCs characterized by an enlarged actin ring, indicating that the alternative NF-κB pathway controls not only OC differentiation but also resorptive activity. Thus, inhibition of NIK is a promising therapeutic strategy for preventing pathological bone loss, while activation of NIK, such as might occur with cIAP antagonists, may accelerate bone loss due to OC activation.

## Results

### Expression of stabilized NIK in OCs leads to decreased bone mass

**Figure 1:**
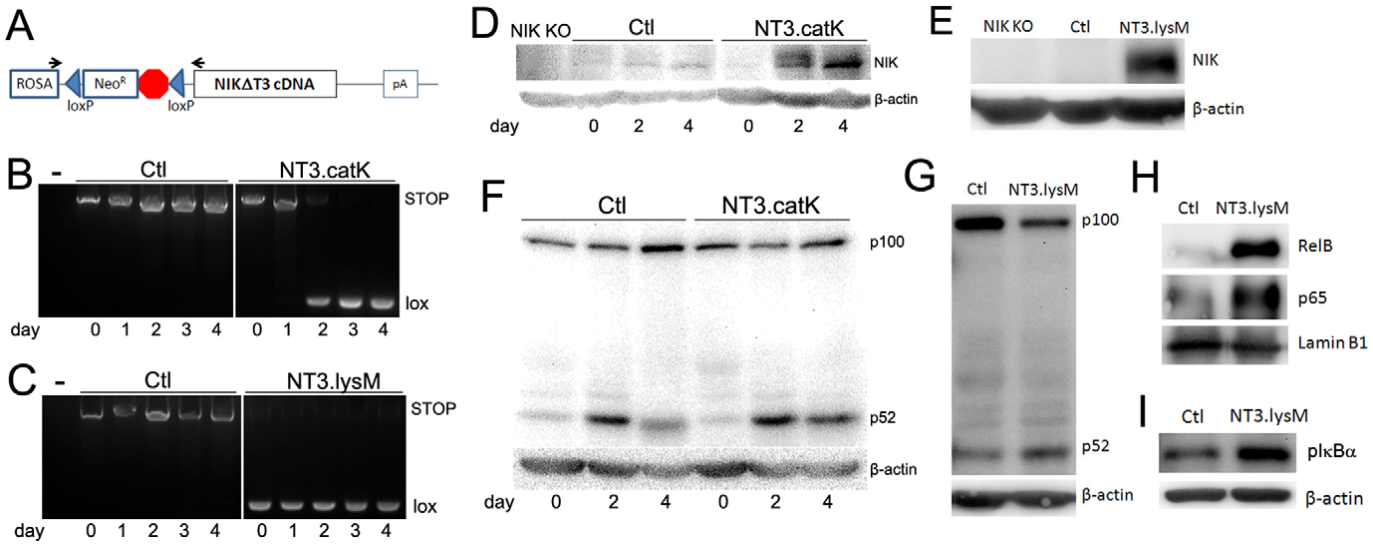
Expression of NIKΔT3 induced by both catK-Cre and lysM-Cre. **A**. Diagram of NT3 locus. NIKΔT3 (NT3) cDNA was knocked into the ROSA26 locus, flanked upstream by a loxP- Neo^R^.STOP-loxP cassette. Cre-mediated loxP recombination removes the Neo^R^.STOP sequence, putting NT3 under control of the ROSA 26 promoter. Black arrows indicate the pair of primers used to amplify across the loxP. **B–C**. Genomic DNA amplification across the loxP sites using NT3.catK (B) and NT3.lysM (C) BMMs cultured with 20 ng/ml RANKL for up to 4 days. ∼3kb STOP and ∼300bp lox bands are generated before and after Cre-mediated deletion, respectively. The left most lanes (-) are negative controls with no genomic DNA in PCR reactions. **D–E**. Western blot analysis of NT3 expression in total cell lysates, using BMMs cultured as in B. NIK KO BMMs serve as a negative control. **F–G**. p100/p52 processing was assessed by Western blot in total cell lysates from Ctl and NT3.catK BMMs cultured in RANKL for the indicated times, or in unstimulated NT3.lysM BMMs. **H**. Evaluation of RelB and p65 levels in nuclear extracts of NT3.lysM BMMs. **I**. Western blot analysis of IκBα phosphorylation in NT3.lysM BMM total lysates.

We obtained transgenic mice in which mutant NIK lacking the TRAF3 binding domain (aa78–84) was knocked into the ROSA26 locus, flanked upstream by a loxP-Neo^R^.STOP-loxP cassette (Fig 1A) [15]. This NIKΔTRAF3 (hereafter referred to as NT3) protein cannot bind to the TRAF3/TRAF2/cIAP complex and is stabilized, even in resting cells. In order to target NT3 to the osteoclast lineage, we mated homozygous NT3 mice to heterozygous cathepsin K (catK)-Cre and lysozyme M (lysM)-Cre lines, both of which have previously been shown to mediate deletion of floxed alleles in OCs [16]–[21]. In each case, all of the progeny had one NT3 allele and half were Cre+ driving expression of NT3 in the OC lineage. We refer to Cre+ progeny from catK-Cre matings as NT3.catK, and those from lysM-Cre matings as NT3.lysM. Neither catK-Cre nor lysM-Cre heterozygous mice have detectable OC phenotypes. Therefore, we used Cre negative littermates as controls (Ctl) in all experiments. PCR analysis of genomic DNA isolated from cultures of bone marrow macrophages (BMMs) treated with RANKL, amplifying across the loxP sites, demonstrates that NT3.catK precursors deleted the STOP sequence beginning on day 2 of RANKL treatment, as seen by the appearance of the smaller amplicon (Fig 1B). NT3.lysM BMMs have completely deleted the STOP before exposure to RANKL (Fig 1C). Immunoblots show an increase in NIK protein that corresponds with the Cre-mediated deletion of the STOP sequence (Fig 1D, E). Congruently, p52 levels are increased at day 4 in NT3.catK cultures (Fig 1F), and in unstimulated NT3.lysM BMMs (Fig 1G). Levels of nuclear RelB are also elevated in NT3.lysM cells (Fig 1H). Thus, NT3 expression stabilizes NIK and drives processing of p100 to p52 and nuclear translocation of RelB, indicating activation of alternative NF-κB. Nuclear p65 levels and phosphorylation of IκBα are also enhanced in NT3.lysM BMMs, compared to Ctl (Fig 1H,I), demonstrating that classical NF-κB is also activated by the transgene.

**Figure 2:**
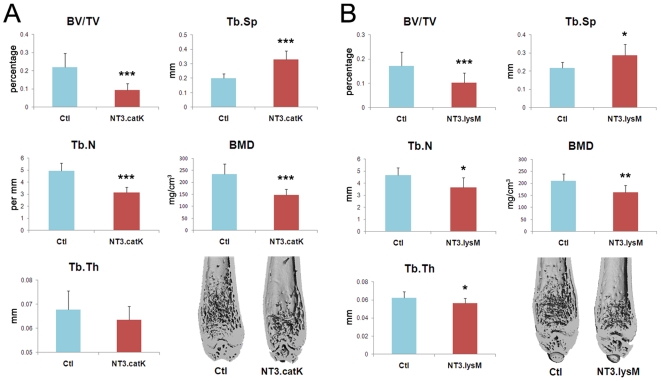
Decreased bone mass in both NT3.catK and NT3.lysM in vivo. Left femurs of 8 weeks old mice from NT3.catK (**A**) and NT3.lysM (**B**) mice and their littermate controls (Ctl) were scanned by microCT. BV/TV (bone volume per tissue volume), Tb.N (trabecular number), Tb.Th (trabecular thickness), Tb.Sp (trabecular spacing), and BMD (bone mineral density) were analyzed. * *p<0.05*; ** *p<0.01*; *** *p<0.001* versus controls., n = 16 NT3.catK; n = 10 Ctl; n = 6 NT3.lysM; n = 10 Ctl.

**Figure 3:**
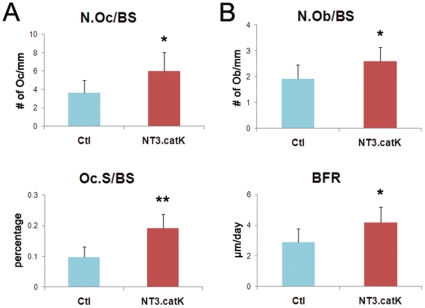
Histomorphometry analysis of NT3.catK mice. 8 week old NT3.catK mice were labeled calcein 9 and 2 days prior to sacrifice. Left tibias were fixed and sectioned for histomorphometric analysis. **A**. Osteoclast-related parameters, N.Oc/BS (number of osteoclasts per bone surface) and Oc.S/BS (osteoclast surface per bone surface), are shown. **B**. Osteoblast-related parameters include N.Ob/BS (number of osteoblasts per bone surface) and BFR (expressed as bone formation rate per bone surface). * *p*≤*0.05*; ** *p*≤*0.01* versus controls. n = 6 Ctl; n = 4 NT3.catK.

**Figure 4:**
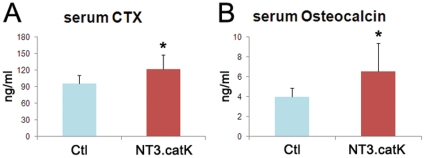
Serum markers of bone resorption and formation activity in NT3.catK mice. Serum from 8-week old control and NT3.catK mice were collected and subject to CTX (**A**) and osteocalcin (**B**) assay. * *p<0.001* versus controls. n = 21 Ctl; n = 17 NT3.catK.

We next analyzed the bone phenotype of NT3.catK and NT3.lysM mice at 8 weeks of age by microCT, compared to age and sex matched Ctl mice. Both NT3 lines had a significantly lower trabecular bone volume (BV/TV), decreased by 57% in NT3.catK and by 40% in NT3.lysM. Bone mineral density (BMD) was less, accompanied by increased trabecular spacing and decreased trabecular numbers, indicating decreased bone mass in the trabecular compartment of NT3 mice (Fig 2). Cortical bone area was also significantly decreased in NT3.catK mice (not shown). In order to determine how the OC and osteoblast (OB) compartments were affected by the transgene, we examined NT3.catK tibias by histomorphometry. Both the number of OCs and the trabecular bone surface covered by OCs was increased to approximately double that of Ctl (Fig 3A). Despite an absence of transgene expression in OBs in NT3.catK mice, the number of OBs and the bone formation rate were also significantly elevated compared to Ctl (Fig 3B), likely reflecting coupling of OC and OB activities. Supporting these histomorphometric results, serum markers of both OC and OB activity were also increased in the 8 week old NT3.catK mice (Fig 4). Thus, constitutive expression of NIK in the OC lineage leads to high turnover osteoporosis.

### Constitutive NIK activation enhances osteoclastogenesis and bone resorption in vitro

**Figure 5:**
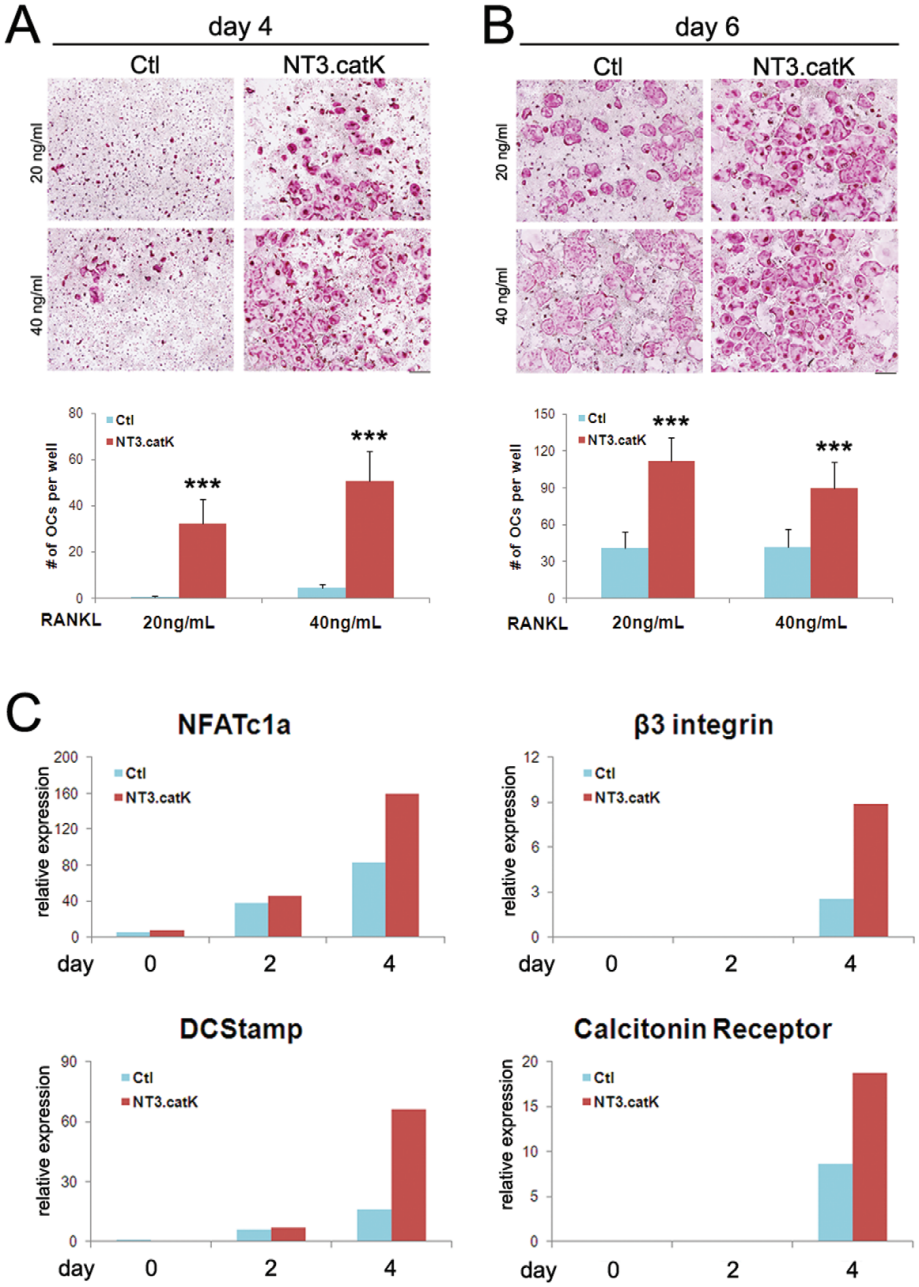
Increased osteoclastogenesis of NT3.catK BMMs in vitro. **A–B**. Control and NT3.catK BMMs were incubated with 20 or 40 ng/ml of RANKL. Cells were fixed and TRAP-stained at day 4 (A) and day 6 (B). TRAP-positive multinucleated cells in each well were counted, n = 6–9. * *p<0.05*; ** *p<0.01* versus Ctl. Scale bar indicates 500 µm. **C**. Ctl and NT3.catK BMMs were cultured with 20 ng/ml RANKL, and RNA was prepared on day 0, 2, and 4, to monitor the transcription levels of indicated OC differentiation markers by quantitative real time RT-PCR, normalized to levels of *Gapdh*.

To determine the effect of NT3 on osteoclastogenesis in vitro, NT3.catK BMMs were cultured in various doses of RANKL, then stained for tartrate resistant acid phosphatase (TRAP), a marker of OC differentiation. The greatest difference between the transgene positive and Ctl cultures was at suboptimal doses (20–40 ng/mL) of RANKL. At these doses, NT3.catK multinucleated TRAP+ OCs formed earlier than Ctl, and at both 4 and 6 days of culture they also formed more OCs (Fig 5A,B). Similarly, NT3.lysM BMMs also form more OCs than controls at low RANKL doses (21±5 vs.12±2at 20 ng/ml; 40±5 vs 17±3 at 40 ng/ml; p<0.05 for both doses). At higher doses, similar numbers of confluent OCs formed, although this occurred earlier in NT3-expressing cultures (not shown). Quantitative RT-PCR analysis demonstrated that the differentiation markers NFATc1, β3 integrin, DC-Stamp and calcitonin receptor were all induced to higher levels in the presence of the NT3 transgene, in cells cultured with 20 ng/ml RANKL (Fig 5C).

**Figure 6:**
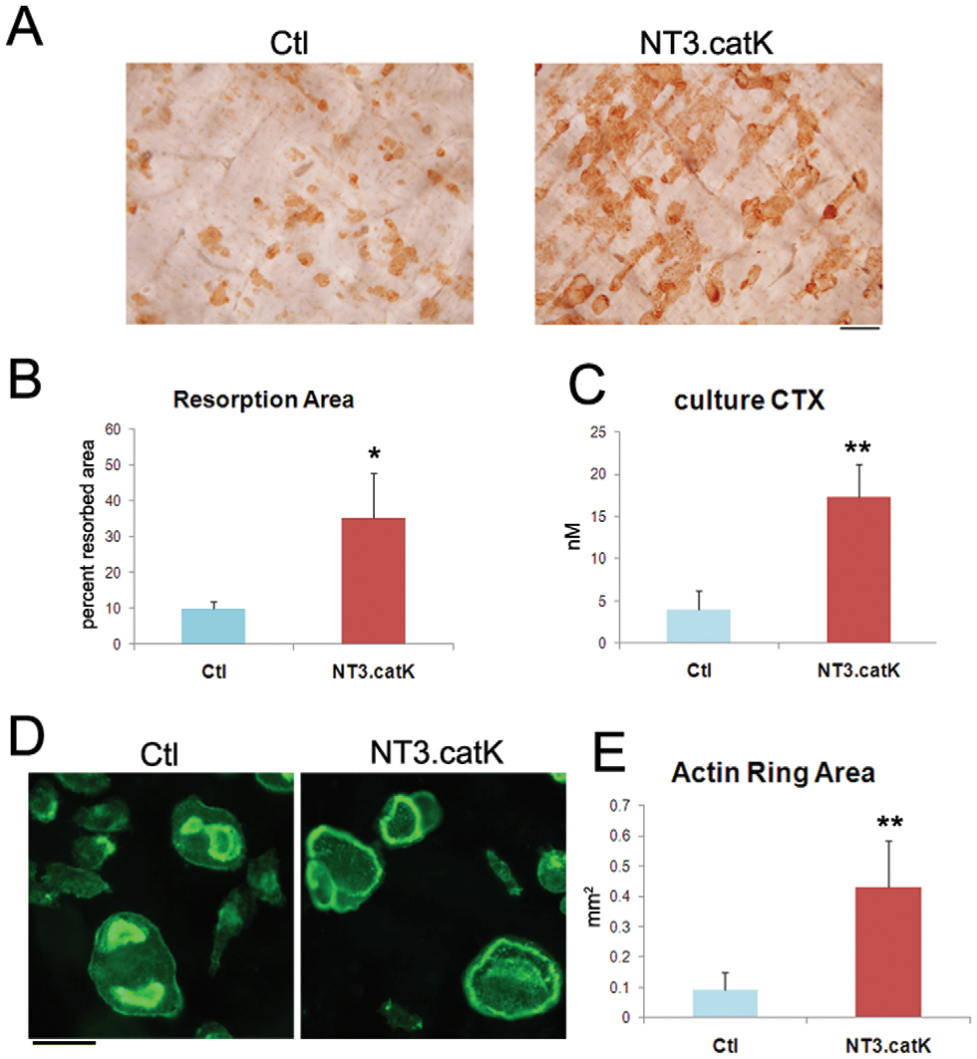
Increased activity of NT3.catK osteoclasts in vitro. **A–B**. Control and NT3.catK OC precursors were cultured with 20 ng/ml RANKL on bone slices for 10 days. (A)Bone slices were fixed and stained with peroxidase-conjugated wheat germ agglutinin to visualize the resoprtion pits. (B) Pit area was quantitated on bone slices from A. n = 3 per condition. **C–E**. Preosteoclasts were generated on plastic using 100 ng/ml RANKL for 2 days, then lifted and replated on bone for culture with 100 ng/ml RANKL for an additional 2 days. (C) Media was collected for culture CTX assay. n = 3. (D) Bone slices were fixed and stained with Alexa 488 phalloidin to visualize actin rings. Scale bar, 100 µm. (E) Actin ring area was quantified. * *p<0.05*; ** *p<0.01* versus controls.

When OC precursors were cultured on bone slices for 10 days in 20 ng/ml RANKL, NT3.catK cultures showed more than 3-fold more resorbed area than Ctl cultures (Fig 6A). In order to determine whether NT3.catK OCs had increased activity per cell, we used a short-term resorption assay in which equal numbers of preosteoclasts, generated on plastic with RANKL for 2 days, were plated on bone slices for 2 more days, and resorption was assessed by measurement of CTX released into the media. Cells were cultured in 100 ng/ml RANKL, a dose at which osteoclastogenesis was comparable in NT3.catK and Ctl cultures, as determined by counting the number of OCs with actin rings on each bone slice. In this assay, CTX release was again more than 3-fold higher in NT3-expressing cells (Fig 6B). The actin ring is the critical cytoskeletal structure required for an OC's resorptive ability. We therefore stained the OCs on bone with Alexa488-phalloidin, and found that the actin rings of NT3-catK cells were significantly larger than Ctl (Fig 6C). These results suggest that the alternative NF-κB pathway controls not only OC differentiation, but also OC activity.

### NT3.catK mice have increased inflammatory osteolysis

**Figure 7:**
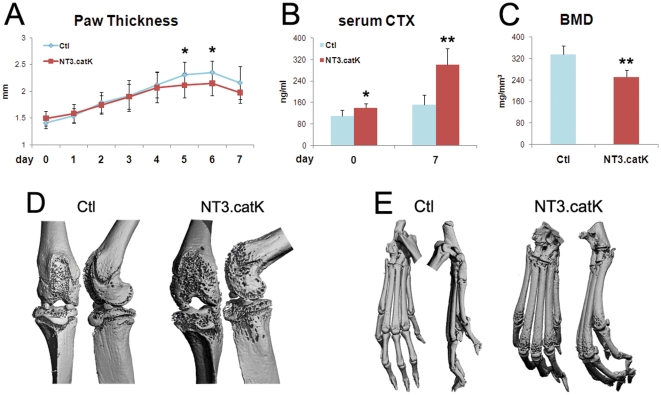
Increased bone erosion in NT3.catK mice during serum transfer arthritis. **A**. Hindpaw thickness was measured bilaterally every day following serum injection on day 0 and 2. n = 8 Ctl; n = 12 NT3.catK. **B**. Serum was collected on day 0 and 7 and used for CTX assay to measure OC activity. n = 5 per group. **C–E**. Mice were sacrificed on day 7, and hind legs were fixed and scanned with microCT. (C) BMD of the distal femur and proximal tibia, including both cortical and trabecular bone. n = 5 per group. 3D reconstructions of knee joints (D) and paws (E) from both front and side views are displayed. * *p<0.05*; ** *p<0.01* versus controls.

Serum transfer arthritis (STA), is a well-characterized model of inflammatory bone loss that mimics rheumatoid arthritis [22]. In STA, serum from arthritic K/BxN mice is injected intraperitoneally into naïve mice, causing joint-centered inflammation and local OC-mediated bone erosion. We injected 8-week old NT3.catK and Ctl littermates with serum on days 0 and 2, then followed the inflammatory response by measuring hindpaw thickness. Bone resorption was assessed by measurement of serum CTX and imaging of joints by microCT. Both groups responded with paw swelling, although the peak at day 5–6 was slightly lower in the NT3-expressing group (Fig 7A). Despite this small decrease in inflammation, the level of bone resorption was twice as high in NT3.catK mice compared to Ctl, as shown by serum CTX levels (Fig 7B). MicroCT analysis of the paws and knee joints also showed increased erosion of cortical bone in NT3.catK mice (Fig 7C–E). Thus, expression of activated NIK only in the OC lineage enhances the osteolytic response to inflammation.

## Discussion

Unlike the classical NF-κB pathway, which is activated by a wide array of inflammatory and infectious stimuli, the alternative NF-κB pathway is activated by only a small subset of cytokines, including RANKL, Ltβ, CD40L and BAFF [5]. Knockouts of various alternative pathway components have demonstrated roles in maturation of B cells [23] and induction of TH17 cells [24], as well as differentiation of osteoclasts [8], suggesting that this pathway might be a relatively specific target for autoimmune diseases, especially those associated with bone loss. The stability of NIK is one of the key control points for activation of the alternative NF-κB pathway. Recent studies of mutations in multiple myelomas revealed that NIK, or more frequently proteins such as TRAF3 and cIAPs that control NIK degradation, are targets for mutation in these tumors [13], [14]. This observation led to generation of transgenic mice expressing a stabilized form of NIK, using a tissue-specific Cre-mediated activation approach, which results in tissue-specific constitutive alternative pathway activation [15]. By expressing this NT3 transgene in the OC lineage, we now describe the effects of NIK activation on bone homeostasis and inflammatory bone loss.

In order to express NT3 in OCs, we utilized two Cre transgenic lines that have previously been shown to delete floxed alleles in this lineage. LysM-Cre mediates deletion in neutrophils, macrophages, and OCs [16], while CatK-Cre is more specific to the OC lineage, deleting at the preosteoclast stage [19]. We found that both NT3.lysM and NT3.catK mice had severe osteoporosis at 8 weeks of age, with an approximate 50% loss in trabecular bone volume. In vitro, osteoclastogenesis occurred at lower doses of RANKL in both NT3.lysM and NT3.catK BMMs, even though NT3.catK BMMs did not show expression of NT3 until they had been cultured in RANKL for 2 days. Nevertheless, even in low doses of RANKL, only an additional 2 days were sufficient for full OC differentiation in NT3.catK cells. Overall, NT3.catK BMMs differentiated more quickly and at lower doses of RANKL than Ctl BMMs, and expressed higher levels of OC differentiation markers NFATc1, β3 integrin, DCStamp and calcitonin receptor. The similarity in phenotypes, in vivo and in vitro, between the earlier expression of NT3 in the lysM line and the later activation in the catK line suggest that the major effects of the alternative pathway occur after commitment to the OC lineage.

In addition to finding increased OC differentiation, our in vitro experiments indicated that NIK activation also enhances the resorptive capacity of each OC. When we plated equal numbers of preosteoclasts on bone at high doses of RANKL, which further normalizes the numbers of OCs formed, we still saw a large increase in bone resorption by NT3-expressing OCs. Intriguingly, actin rings were 3-fold larger in area in NT3.catK OCs compared to Ctl OCs, extending almost to the cell edge. Preliminary experiments suggest that even on plastic, NT3 drives increased expression of a subset of cytoskeleton-associated proteins (J.L. Davis and D.V. Novack, unpublished data). Although there are a number of suggestions in the literature that the cytoskeleton can alter signaling pathways including NF-κB [25]–[27], there are no direct studies indicating that the alternative pathway can regulate the cytoskeleton. Once the actin ring and sealing zone are formed, the OC must secrete enzymes and acid to accomplish bone resorption. Our observation that NT3 OCs are highly efficient bone resorbers may indicate NIK also plays a role in these processes.

Exposure of OC precursors to RANKL causes NIK-dependent processing of p100 to p52, an event necessary for OC differentiation in vitro [8]. Expression of the stabilized form of NIK, NT3, increased this processing event and enhanced osteoclastogenesis. However, we do not know whether this traditional role for NIK is responsible for all of the observed stimulatory effects on OCs. Ablation of NIK causes accumulation of p100 which inhibits classical NF-κB signaling by binding p65, although this does not contribute significantly to the observed block in OC differentiation [6], [8]. Nevertheless, activation of the classical pathway by NT3 could contribute to increased OC numbers, either by supporting the differentiation program or inhibiting apoptosis. In other cell types, NIK has been shown to impact STAT3 signaling [24], [28] and ERK activation [15], [29], and activation of these pathways could impact the OC as well. Further studies will be required to determine all of the pathways impacted by NIK stabilization in the OC.

The most common form of systemic bone loss is postmenopausal osteoporosis caused by estrogen deficiency. In the early phases of this disease, the activity of both OCs and OBs is increased leading to high bone turnover. However, bone resorption and formation are not balanced, and the net effect is bone loss, primarily in the trabeculae [1]. Most studies have focused on stimulation of OCs as the primary event. In physiologic bone remodeling, the differentiation and activity of OBs is coupled to that of OCs via as yet incompletely defined factors, and in early osteoporosis the OB activation is also thought to be secondary to the OC. Analysis of NT3.catK mice demonstrated that, at baseline, the number of OCs was increased, as were serum levels of CTX, a marker of OC activity, indicating that bone resorption was increased. OB number and bone formation rate were also elevated along with osteocalcin, a serum marker of OB activity. This phenotype of low bone mass with elevated OC and OB activity is known as high turnover osteoporosis and mimics the early postmenopausal state.

Another common context for pathological bone loss is rheumatoid arthritis, in which inflammatory mediators such as TNFα promote OC differentiation and activation leading to local osteolysis. We have previously shown that NIK−/− mice are resistant to periarticular bone erosion in the STA model of rheumatoid arthritis, in the context of normal inflammation, suggesting that NIK is important for the osteolytic response itself [12]. We now find that the constitutive NIK activation in NT3.catK mice leads to an exaggerated osteolytic response to STA. MicroCT scanning shows increased pitting of cortical bone around the joints leading to a measurable decrease in overall BMD at the knee. In this model, bone resorption is stimulated due to upregulation of RANKL and TNFα in the joints. Given the enhanced sensitivity of NT3-expressing OC precursors to RANKL, it is not surprising that there is increased bone erosion in vivo. Additionally, NT3.lysM precursors are capable of differentiation to OCs in vitro with TNF alone, whereas Ctl cultures are not (C. Yang and D.V.Novack, unpublished). Combined, our studies of the effects of NIK loss and gain of function indicate that NIK is a key regulator of osteolysis in the context of inflammatory arthritis and represents an attractive therapeutic target.

In addition to its stimulation by pathological conditions such as arthritis, NIK can also be activated pharmacologically. Deletion of the TRAF3 binding domain in NIK blocks its degradation by preventing cIAP-mediated ubiquitination. Therefore, another way to stabilize NIK protein is to block cIAPs directly with small molecule cIAP antagonists [30], [31]. In addition to their role as inhibitors of the alternative NF-κB pathway, cIAPs also have important pro-survival functions downstream of TNF, and inhibition of cIAPs in many tumor cell types leads to apoptosis [32]. Thus, cIAP antagonists are being investigated as anti-tumor therapeutics. Our finding that NIK stabilization causes low bone mass in growing mice and enhances inflammatory osteolysis raises caution that cIAP antagonist drugs may have significant side effects in bone due to OC activation.

## Materials and Methods

### Reagents and transgenic mice

Macrophage-colony stimulating factor (M-CSF), in the form of CMG 14-12 supernatant, and glutathione-S-transferase RANKL (GST-RANKL) were made as described previously [33], [34]. NIKΔT3 transgenic mice bearing cDNA for NIK lacking the TRAF3 binding domain (NIKΔT3) inserted into the ROSA26 locus [15] and homozygous for the transgene were mated to heterozygous lysozyme M Cre [16] or cathepsin K Cre [19] in a specific pathogen-free facility. All experimental protocols were approved by the Institutional Animal Studies Committee at Washington University School of Medicine, permit number 20080131.

### Osteoclast culture

Bone marrow macrophages (BMMs) were isolated and cultured as described [8]. BMMs were plated in the indicated doses of GST-RANKL in the presence of 1∶25 dilution of CMG 14-12 supernatant as a source of M-CSF, with media changes every 2 days, at the following cell densitities: 7.5×10^3^ for 96-well plates, 2×10^4^ for 48-well plates, for plating on bone slices, or 1.2×10^6^ for 10 cm^2^ dishes. OC cultures were fixed in 3.7% formaldehyde and 0.1% Triton X-100 for 5 minutes, and stained for tartrate resistant acid-phosphatase (TRAP) according to the manufacturer's instructions (Sigma-Aldrich). Total cell lysates were prepared and subjected to immunoblot analysis as described [6]. Antibodies to NIK and p100/p52 were from Cell Signaling Technologies.

### Genomic DNA preparation and PCR

DNA was extracted from cells using the XNAT REDExtract-N-Amp Tissue PCR kit (Sigma-Aldrich), and PCR was performed using the included PCR ReadyMix. Primers used to amplify across the loxP-Neo^R^-STOP-loxP cassette were 5′-TAGGGCGCAGTAGTCCAGGGTTTCC-3′ and 5′-CATCACGGCCTTGTCGTCATCG-3′.

### Real time RT-PCR

Using the NucleoSpin RNA II kit (Machery-Nagel), total RNA was extracted from cells. cDNA was generated from 1 µg total RNA using Sprint RT Complete PCR tubes (Clontech) according to the manufacturer's instructions. RT-PCR was performed on an ABI7300 Real-Time PCR system (Applied Biosystems) using SYBR Advantage premix (Clontech) and the following sets of primers: *NFATc1a*, 5′-GGTAACTCTGTCTTTCTAACCTTAAGCTC-3′ and 5′-GTGATGACCCCAGCATGCACCAGTCACAG-3′; *β3-integrin*, 5′-TGGTGCTCAGATGAGACTTTGTC-3′ and 5′-GACTCTGGAGCACAATTGTCCTT-3′; *DC Stamp*, 5′-ACAAACAGTTCCAAAGCTTGC-3′ and 5′-TCCTTGGGT-TCCTTGCTTC-3′; *Calcitonin Receptor*, 5′-CAAGAACCTTAGCTGCCAGA-3′ and 5′-AAGACGCGGACAATGTTG-3′; *Gapdh*, 5′-CTTCACCACCATGGAGAAGGC-3′ and 5′-GACGGACACATTGGGGGTAG-3′. The amplification reaction was performed for 40 cycles with denaturation at 95°C for 5 seconds, and annealing/extension at 60°C for 31 seconds. Melt curve analysis was performed after each run. The relative abundance of each target was calculated as 1000×2^−(C*t* target gene−Ct Gapdh)^, where *Ct* represents the threshold cycle for each transcript, and *Gapdh* is the reference.

### Bone resorption pit and actin ring staining

To visualize resorption pits, pre-osteoclasts were plated on bovine cortical bone slices and allowed to differentiate in the presence of RANKL and M-CSF. On the desired day, osteoclasts were removed from the bone slices using a cotton swab/soft brush and rinsed with PBS. The slices were then incubated at 37°C with 20µg/mL peroxidase-conjugated wheat germ agglutinin (Sigma-Aldrich) for 30 minutes. The slices were rinsed again with PBS, and then 3,3′-diaminobenzidine was added and the slices were incubated again for 30 minutes. Slices were then rinsed with PBS, dried, and imaged for quantification of pit area.

For analysis of actin rings, bone slices were fixed with 4% ultra-pure paraformaldehyde (Polysciences) and 0.2% Triton X-100 for 10 minutes, then washed with PBS 3 times. Slices were then incubated with Alexa 488-phalloidin (Invitrogen) in the dark for 15 minutes and mounted on microscope slides. Composite pictures were assembled in Photoshop CS2 (Adobe Systems Incorporated) and were imported into BioQuant OSTEO 2010 (BioQuant Image Analysis Corporation) for quantification of pit or actin ring area.

### CTX (Carboxy-Terminal Collagen Crosslinks) and Osteocalcin Assay

Prior to bleeding, mice were starved of food, but not water, overnight. The amount of type I collagen released into serum was measured using RatLaps™ RIA for serum (Immunodiagnostic Systems Ltd.). Serum osteocalcin was measured by sandwich ELISA (Biomedical Technologies, Inc.). The culture media from bone slices (representing 48 hours resorption) was collected and CTX levels determined with CrossLaps for cell culture (Immunodiagnostic Systems Ltd.) according to the manufacturer's instructions.

### MicroCT analysis

Femurs or tibias were dissected out from sacrificed mice and fixed with formalin overnight. These bones were scanned in μCT40 microCT (Scanco Medical) at 55 kVp, 145 µA, and 16 µm resolution. Gauss sigma of 1.2, Gauss support of 2, lower threshold of 237, and upper threshold of 1000 were used for all the analysis. Regions of interest (ROIs) were selected 50 slices above the growth plate of the distal femur to evaluate the trabecular compartment. ROIs of 180 slices above and below the knee joint, including both cortical and trabecular compartments of distal femur and proximal tibia, were used to assess the extent of bone erosion in the serum transfer arthritis model.

### Histomorphometric analysis

To assess bone formation, calcein (Sigma-Aldrich) was intraperitoneally injected into mice at a dose of 10mg/kg body weight on 2 and 9 days before sacrifice. Dissected femurs and tibias were fixed with formalin. Bones were embedded in methymethacrylate for sectioning, and then were stained for TRAP or left unstained for analysis of calcein labels. Histomorphometric analysis was performed by an observer blinded to genotype using standard parameters and BioQuant OSTEO 2010 software (BioQuant Image Analysis Corporation).

### Serum Transfer Arthritis

Arthritis was induced by intraperitoneal injection of 250 µl K/BxN serum at days 0 and 2 [22], with serum harvested prior to injection on day 0 and at sacrifice on day 7. Ankle thickness in the hind paws was measured daily by dial gauge (Mitutoyo).

### Data Analysis

Values reported graphically are expressed as mean ± SD, with numbers of samples indicated in figure legends. A p-value was obtained through the use of unpaired 1-tailed Student's t-test. *p* values are indicated in each figure, and values less than 0.05 were considered significant.
